# Anti-inflammatory effects of cyclodextrin nanoparticles enable macrophage repolarization and reduce inflammation

**DOI:** 10.1186/s11671-024-04175-6

**Published:** 2024-12-21

**Authors:** Felix E. B. Brettner, Stefanie Gier, Annika Haessler, Jonas Schreiner, Sarah Vogel-Kindgen, Maike Windbergs

**Affiliations:** Institute of Pharmaceutical Technology, Goethe-University Frankfurt, Max-von-Laue-Straße 9, 60438 Frankfurt am Main, Germany

**Keywords:** Nanoparticles, Amphiphilic cyclodextrins, Macrophages, Inflammation, Inflammasome, Lipidomic profiling, Immunomodulation, Anti-inflammatory effect

## Abstract

Inflammation plays a critical role in the pathophysiology of many diseases, and dysregulation of the involved signaling cascades often culminates in uncontrollable disease progression and, ultimately, chronic manifestation. Addressing these disorders requires balancing inflammation control while preserving essential immune functions. Cyclodextrins (CDs), particularly β-CD, have gained attention as biocompatible biomaterials with intrinsic anti-inflammatory properties, and chemical modification of their backbone offers a promising strategy to enhance their physicochemical properties, adaptability, and therapeutic potential. This study evaluated and characterized the immunomodulatory effects of amphiphilic CD derivatives, which self-assemble into nanoparticles, compared to soluble parent β-CD. In a human macrophage model, CD nanoparticles demonstrated superior anti-inflammatory activity, with derivative-specific effects tied to their physicochemical properties, surpassing the soluble β-CD control. Alongside the downregulation of key pro-inflammatory markers, significant reductions in inflammasome activation and changes in lipid profiles were observed. The findings of this study underscore the potential of cyclodextrin-based nanoparticles as versatile biomaterials for treating the complex pathophysiology of various acute and chronic inflammation-associated disorders.

**Supplementary Information:**

The online version contains supplementary material available at 10.1186/s11671-024-04175-6.

## Introduction

Inflammation is a complex physiological response of the immune system to harmful stimuli, such as tissue injury or infection by pathogens. It is characterized by highly coordinated physiological and biochemical events involving various immune cells, chemical mediators, and molecular pathways that aim to protect the body, eliminate exogenous harms, and facilitate tissue regeneration. Thus, inflammation plays a crucial role in establishing cellular homeostasis and is an integral function of the human body [1–3]. While an essential defense mechanism, excessive or chronic inflammation can lead to severe organ or tissue damage and contribute to the development of multiple disorders, including autoimmune diseases as well as neurodegenerative, cardiovascular, and rheumatic conditions [4–7]. Current strategies to advance therapeutic approaches involve applying suitable biocompatible materials, and the identification and design of biomaterials that can directly interact with cells and tissue of the inflamed microenvironment is in high demand [8–12]. As cells of the innate immune system, macrophages are particularly attractive targets for immune modulation via biomaterials due to their unique role in tissue repair and homeostasis regarding all stages of inflammation [13–16]. In this context, naturally derived cyclodextrins (CDs) are of considerable interest, as current research data hints at intrinsic anti-inflammatory properties of native β-CD and water-soluble CD derivatives [17, 18]. In general, CDs harbor distinctive stereochemical features due to the circular α−1,4 glycosidic linkage of D-glucopyranose units, forming a truncated cone shape with the primary hydroxyl groups at the narrow rim and the secondary hydroxyl groups at the wider edge [19, 20]. This molecular structure results in a relatively hydrophobic inner cavity and a hydrophilic outer surface, which enables the formation of inclusion complexes trapping various small molecules and even larger biomacromolecules into a hydrophobic core [21–25]. Naturally occurring CDs are classified as α-, β-, and γ-CDs depending on the number of connected D-glucopyranose with six, seven, and eight sugar units, respectively [26–28]. In addition to their remarkable molecular features, native hydrophilic CDs are non-toxic and hold the GRAS ("Generally Recognized as Safe") status [29]. All these characteristics facilitate the use of CDs in various fields, including pharmaceutical and biotechnological sciences, as well as food, cosmetics, and agriculture. Especially β-CDs are widely used to enhance the solubility, stability, or bioavailability of poorly soluble drugs without toxic side effects [21, 30, 31]. Moreover, the unique molecular structure of CDs is predestined for chemical alteration through the addition of functional groups, selectively altering the physico- and biochemical characteristics of the resulting derivatives. Chemically modifying the hydrophilic core structure by grafting aliphatic chains, for example, renders the CD molecules amphiphilic, initiating the surfactant-free self-assembly into vesicles, micelles, or nanoparticles (NPs) [32–35]. Via adaption of the chain length or further chemical alterations, the characteristics of these supramolecular structures can be specifically tailored to meet the particular requirements of the desired biomaterial [36]. While various studies have already shown the applicability of CD-based nanoparticles in drug delivery, the full potential of such systems regarding their inherent material properties has not yet been adequately addressed [17, 18, 37]. In this study, we investigated the intrinsic immunomodulatory characteristics of three β-CD-based nanoparticle (CD-NPs) types with a particular focus on the influence of their different physicochemical properties and in direct comparison to the soluble parent cyclodextrin. Using a human pro-inflammatory-primed macrophage model, the effects of the NPs on classical inflammation markers were evaluated on mRNA and protein levels. In addition to molecular biological analyses, confocal laser scanning and label-free selective Raman microscopy were applied to further elucidate cellular responses upon interaction with CD-based nanoparticles.

## Materials and methods

### Materials

Unless otherwise stated, all materials were purchased from Merck (Taufkirchen, Germany). All secondary antibodies and fluorescent dyes were obtained from Thermo Fisher Scientific (Waltham, MA, USA). Primary antibodies were purchased from BIOMOL GmbH (Hamburg, Germany).

### Cyclodextrin nanoparticle preparation

Heptakis-6-*O*-hexanoyl-β-cyclodextrin (CDOC_6_), heptakis-6-*O*-lauroyl-β-cyclodextrin (CDOC_12_), and heptakis-(6-deoxy-6-hexylthio)-β-cyclodextrin (CDSC_6_) for the nanoparticle preparation were synthesized as previously described, and all nanoparticle types were prepared by nanoprecipitation [36]. Briefly, the amphiphilic cyclodextrin derivatives were dissolved in acetone (CDOC_6_ and CDOC_12_) or tetrahydrofuran (CDSC_6_) and dropped into an aqueous phase of purified water under constant stirring at room temperature (RT); the flow rate was set to 5 ml h^−1^ and controlled by a syringe pump (Pump 11 Pico Elite Plus, Harvard apparatus, March-Hugstetten, Germany). The aqueous/ organic phase ratio was 2:1 (v/v) for CDOC_6_, and CDSC_6_, and 8:1 (v/v) for CDOC_12_. After nanoprecipitation, the organic solvent was evaporated under reduced pressure. All nanosuspensions were stored at 4 °C until further use.

### Physicochemical nanoparticle characterization

The intensity-based hydrodynamic diameter, the polydispersity index (PDI), and ζ potential of the nanoparticles were regularly monitored at 25 °C by dynamic light scattering (DLS) and electrophoretic light scattering (ELS), ensuring batch consistency (Zetasizer NanoZS, Malvern Panalytical, Malvern, UK). All measurements were carried out in ultrapure water, and the results are expressed as mean ± standard deviation (SD).

### Scanning electron microscopy

Scanning electron microscopy (SEM) was employed to visualize amphiphilic CD nanocarriers with an electron high tension of 25 kV (Zeiss EVO10 microscope, Carl Zeiss AG, Oberkochen, Germany). Sample preparation was performed as previously described [36]. Single droplets of each nanocarrier suspension were applied onto aluminum foil and rapidly frozen in liquid nitrogen. The dispersant was subsequently evaporated under reduced pressure. Finally, the samples were coated with a gold–palladium layer (SC7620 mini Sputter Coater, Quorum Technologies, Lewes, UK).

### Cell culture

The human monocyte cell line THP-1 was purchased from the German Collection of Microorganisms and Cell Cultures GmbH (Braunschweig, Germany). The cells were cultured in Roswell Park Memorial Institute 1640 Medium (RPMI, Gibco Life Technologies, Darmstadt, Germany) supplemented with 10% fetal calf serum (FCS, Lonza, Verviers, Belgium). The medium was changed every two days, and the cells were maintained in a humidified atmosphere at 37 °C and 5% CO_2_.

### Differentiation and polarization of human monocytes into resting and pro-inflammatory macrophages.

To differentiate the THP-1 monocytes into resting macrophages (MΦ0), cells were seeded and treated with 45 ng ml^−1^ phorbol-12-myristate-13-acetate (PMA). After 48 h, medium was removed, and the cells rested for 24 h in fresh RPMI medium to complete the differentiation. The MΦ0 cells were further polarized into pro-inflammatory macrophages (MΦ1) via the addition of 50 ng ml^−1^ lipopolysaccharide from *Escherichia coli* 026:B6 (LPS, Thermo Fisher (Waltham, MA) and 20 ng ml^−1^ human interferon-γ (IFNγ, ImmunoTools, Friesoythe, Germany) for 24 h. The now pro-inflammatory-primed macrophages were used to evaluate the anti-inflammatory effects of the different nanoparticle types. Therefore, the supernatant was discarded, and fresh RPMI medium supplemented with 50 ng ml^−1^ LPS, 20 ng ml^−1^ IFNγ, and either 0.2 mg ml^−1^ or 0.1 mg ml^−1^ nanoparticle suspension was added. Depending on the experimental aim, MΦ1 cells were cultivated for up to 48 h (37 °C, 5% CO_2_). The applied protocol was optimized regarding stimuli concentration and treatment times, ensuring adequate and reproducible cytokine and marker expression for downstream studies.

### Immunocytochemistry

In addition to light microscopy, the successful differentiation of THP-1 cells was evaluated via immunofluorescence. Briefly, 350,000 cells per well (12-well format) were seeded onto coverslips (Ø 18 mm) and treated as mentioned above. Afterward, cells were washed twice with 1 × phosphate-buffered saline (1 × PBS) and fixed for 15 min at RT using a formaldehyde solution (4% (v/v) in 1 × PBS). The fixing solution was discarded, and cells were washed thrice with washing buffer (1 × PBS with 0.05% Tween 20). Subsequently, a solution of 0,165 µM Alexa Fluor 488 Phalloidin diluted in 1 × PBS was added, and samples were incubated for 1 h at RT to stain the cytoskeleton. The cells were washed three times, and cell nuclei were stained with 4′,6-diamidino-2-phenylindole (DAPI, 3 µM in 1 × PBS) for 5 min. After two final washing steps, the coverslips were mounted with FluorSave Reagent and dried overnight. The cells were imaged using a confocal laser scanning microscope (Zeiss LSM 900, Carl Zeiss AG, Oberkochen, Germany).

### Evaluation of biocompatibility

Using the 3-(4,5-dimethylthiazol-2-yl)−2,5-diphenyltetrazolium bromide assay (MTT), potential cytotoxicity of the different nanocarriers was evaluated in both MΦ0 and MΦ1. Therefore, 40,000 THP-1 cells per well were seeded in a 96-well plate, differentiated and polarized, and subsequently incubated with different concentrations of all three nanocarrier types for 24 h and 48 h at 37 °C and 5% CO_2_. Afterward, cells were washed with 1 × PBS, and 1 mg ml^−1^ MTT reagent (diluted in RPMI) was added per well. After incubation for 4 h (37 °C, 5% CO_2_), MTT-containing supernatant was discarded, and the formazan crystals were dissolved in dimethyl sulfoxide. Absorbance measurement was performed at 570 nm (Spark multimode microplate reader, Tecan, Männerdorf, Switzerland). Cells treated with 1% TritonX100 (diluted in RPMI) or solely with RPMI served as the positive and negative control, respectively. The cell viability was calculated according to Eq. 1.

1 $$Cell \,viability \,(\%)= \frac{{Absorbance}_{cells\, incubate\, with\, nanocarriers}-{Absorbance}_{positive\, control}}{{Absorbance}_{negative\, control}-{Absorbance}_{positive\, control}} \times 100$$

### RNA isolation, reverse transcription, and real-time qPCR

Initially, 350,000 THP-1 cells were seeded per well (12-well format), differentiated, and polarized to MΦ1. Subsequently, MΦ1 cells were incubated with the CD nanocarriers for different time points (8 h, 24 h, 48 h). Cells were lyzed through the addition of Tri-Reagent, and total ribonucleic acid (RNA) was extracted with the Direct-zol RNA MiniPrep Plus Kit (Zymo Research Europe GmbH, Freiburg, Germany) following the manufacturer's instructions. RNA concentration and purity were validated photometrically using the NanoQuant Plate of the Spark multimode microplate reader (Tecan, Männerdorf, Switzerland). First-strand synthesis of 500 ng total RNA was conducted using the Maxima H Minus cDNA Synthesis Master Mix Kit (ThermoFisher Scientific, Waltham, MA, USA) according to the manufacturer; concentration was determined photometrically. 10 ng complementary deoxyribonucleic acid (cDNA) was analyzed via the StepOnePlus Real-Time PCR System (Applied Biosystem, Waltham, MA, USA) using SYBR Green PowerTrack (ThermoFisher Scientific, Waltham, MA, USA) as fluorescent dye. The following primer (5′−3′ orientation) were used for amplification: GAPDH *forward*: CGGGAAGCTTGTCATCAATGG, GAPDH *reverse*: GGCAGTGATGGCATGGACTG, CD40 *forward*: CAGCCAGGACAGAAACTGGTGAGT, CD40 *reverse*: CTTCTTCACAGGTGCAGATGGTGTC, CD80 *forward*: CACTTCTGTTCAGGTGTTATCC, CD80 *reverse*: GGTGTAGGGAAGTCAGCTTTG, CD86 *forward*: CTCTTTGTGATGGCCTTCCTG, CD86 *reverse*: CTTAGGTTCTGGGTAACCGTG, TNFα *forward*: CCTCTCTCTAATCAGCCCTCTG, TNFα *reverse*: GAGGACCTGGGAGTAGATGAG, IL1β *forward*: AGCTACGAATCTCCGACCAC, IL1β *reverse*: CGTTATCCCATGTGTCGAAGAA, IL6 *forward*: ACTCACCTCTTCAGAACGAATTG, IL6 *reverse*: CCATCTTTGGAAGGTTCAGGTTG. As a housekeeping gene, GAPDH was used to normalize all measurements, whereas an untreated MΦ1 sample served as control. Additionally, samples of MΦ0 macrophages were routinely analyzed to ensure sample quality. The Ct values were evaluated according to the 2^−ΔΔCt^ method, and results are illustrated as fold change compared to the untreated MΦ1 control [38].

### Cytokine release

The supernatants of treated and untreated MΦ1 were analyzed regarding the release of the cytokines tumor necrosis factor α (TNFα), interleukin 1 β (IL1β), and interleukin 6 (IL6). The cell culture media were collected and centrifuged (13,000×g, 15 min, 4 °C) to separate cell debris; the cell-free supernatant was stored at −80 °C until further use. Cytokine release was quantified using uncoated ELISA kits for the respective cytokines according to the manufacturer (Invitrogen, Waltham, MA, USA). Absorption was measured at 450 nm with reference wavelength at 570 nm (Spark multimode microplate reader, Tecan, Männerdorf, Switzerland); the respective absorbance values were subtracted, plotted against a calibration curve, and the cytokine concentrations obtained were further used for analysis according to Eq. 2.

2 $$Cytokine\, release\,(\%)= \frac{{Cytokine\, conc.}_{CDNP\, treated\, macrophages}-{Cytokine\, conc. }_{blank\, control}}{{Cytokine\, conc. }_{Medium\, treated \,macrophages}-{Cytokine\, conc. }_{blank\, conctrol}} \times 100$$

Samples of untreated MΦ1 macrophages served as control, whereas samples of MΦ0 cells were routinely tested to validate sample preparation and assay reproducibility.

### Inflammasome staining

After treatment of MΦ1 with CDOC_6_, CDOC_12,_ and CDSC_6_ nanoparticles (0.2 mg ml^−1^, 24 h), the subcellular localization of NLRP3 and ASC, two inflammasome components, was evaluated via immunocytochemistry. Untreated MΦ0 and MΦ1 macrophages served as controls. Therefore, cells were grown and differentiated on coverslips; fixation of the cells was conducted as described above. After washing, blocking solution (washing buffer containing 3% BSA) was added for 1 h at RT. Subsequently, a mixture of rabbit anti-ASC IgG (1:200) and mouse anti-NLRP3 IgG 2b (1:200) was applied, and samples were incubated at 4 °C overnight. Samples were washed three times with washing buffer and further incubated with the secondary antibodies goat anti-mouse IgG Alexa Fluor 633 (1:1000), followed by incubation with goat anti-rabbit IgG Alexa Fluor 555 (1:1000) for 1 h. Preliminary experiments and adequate control stainings were performed, optimizing antibody concentrations and excluding cross-reactivity. Afterward, samples were washed thrice, and cytoskeleton and cell nuclei were stained with Alexa Fluor 488 Phalloidin and DAPI, respectively, as written above. After covering the slides in FluorSave Reagent, the cells were imaged using a confocal laser scanning microscope (Zeiss LSM 900, Carl Zeiss AG, Oberkochen, Germany). Using the ImageJ Plugin JACoP for co-localization, quantification was conducted using the Manders' coefficient fraction 1 (M1). All quantification measurements were carried out in triplicate.

### Raman imaging and data processing

For Raman analysis, MΦ1 cells were treated with 0.2 mg ml^−1^ of the different CD nanoparticles for 24 h and subsequently fixed in 4% formaldehyde solution (in 1 × PBS) for 10 min. Untreated MΦ1 and MΦ0 macrophages served as controls. Cells were imaged using a confocal Raman microscope (Alpha300R + , WITec GmbH, Ulm, Germany) equipped with a 532 nm laser set to a power of 37 mW in front of the 63 × water dipping objective (numerical aperture 1.0). The Raman scans were acquired in a range of 400–3720 cm^−1^ with a spectral resolution of 4 cm^−1^, an integration rate of 0.2 s, and a spatial resolution of 0.5 µm. The background of the Raman spectra of each scan was subtracted by using the 'shape' function (filter size: 100) in the Project Four software (WITec GmbH, Ulm, Germany). Cosmic rays were removed using the Project Four software as well. Next, scans were imported to Matlab (Mathworks, Inc., Natick, USA) and separately processed. After smoothing the data with a Savitzky-Golay filter (window size: 9, order: 3) and normalization, the spectral unmixing algorithm N-FINDR was used to identify endmember spectra in the scans. Abundance maps were estimated using the nuclei and lipid endmember signatures for each scan. Next, the 20 most correlated spectra to the respective lipid endmember were subjected to a principal component analysis (PCA). Furthermore, they were used to calculate the mean lipid spectra of each scan. As a final step, Raman images were smoothed using cubic convolution interpolation.

### Statistical analysis

ELISA data, cytotoxicity, and Manders' coefficient 1 evaluation were analyzed regarding statistical significance using One-Way ANOVA followed by the Dunn-Sidak posthoc test. The significance of RT-qPCR data was evaluated via One-Way ANOVA or an unpaired *t*-test, according to the used controls. Data were considered significant if the *p*-value was below 0.05 with *p < 0.05, **p < 0.01, and ***p < 0.001.

## Results

### Formation of cyclodextrin nanoparticles and macrophage polarization

**Fig. 1:**
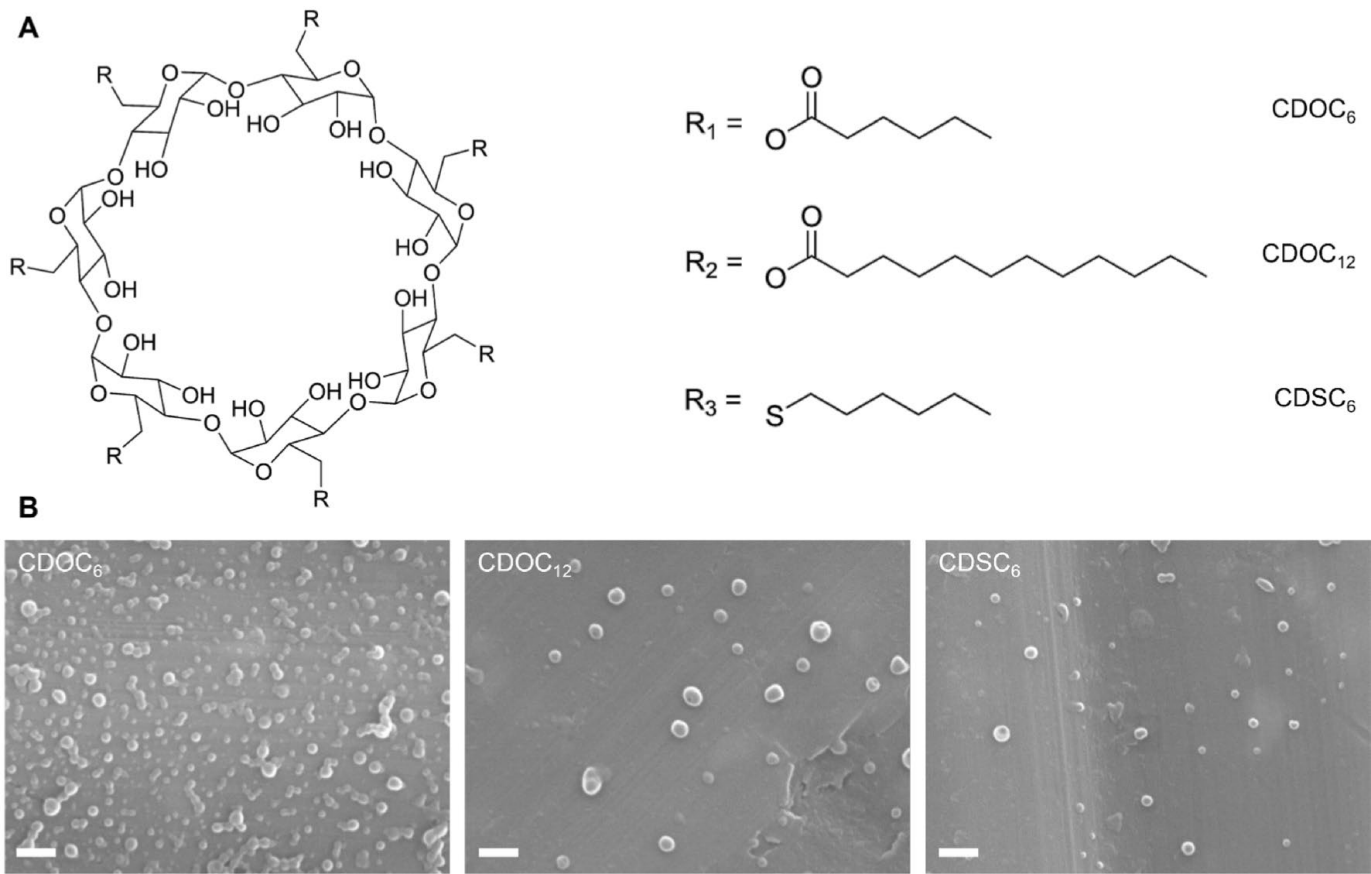
**A** Chemical structure of heptakis-6-O-hexanoyl-β-cyclodextrin (CDOC_6_), heptakis-6-O-lauroyl-β-cyclodextrin (CDOC_12_), heptakis-(6-deoxy-6-hexylthio)-β-cyclodextrin (CDSC_6_) and **B** micrographs at 25,000 × magnification obtained by scanning electron microscopy of CDOC_6_-, CDOC_12_-, and CDSC_6_-based nanocarriers. Scale bars depict 400 nm

The amphiphilic CDs were obtained by linking different aliphatic chains via ester (CDOC_6_ or CDOC_12_) or thioether bonding (CDSC_6_) onto the native β-CD-backbone (Fig. 1A). The derivatives were dissolved in an organic solvent, and particle formation was achieved via nanoprecipitation. All CD derivatives self-assembled into solid, spherical, and monodisperse nanospheres with particle sizes below 250 nm (Fig. 1B).

The nanometer size as well as the negative surface charge of the CD-NPs was confirmed via DLS and ELS measurements. CDOC_12_- and CDSC_6_-based CD-NPs were larger in size and exhibited a slightly higher polydispersity index (PDI) than CDOC_6_-NPs (Table 1). In-depth characterization of the CD derivatives via ^1^H NMR and ATR-IR, as well as stability studies of the respective NPs in biologically relevant medium was previously reported [36].

**Table 1:** Size, polydispersity index (PDI), ζ potential of CD-NPs obtained via DLS and ELS measurements

Formulation	Size [nm]	PDI	ζ potential [mV]
CDOC_6_	141.31 ± 3.99	0.092 ± 0.015	− 33.12 ± 1.07
CDOC_12_	210.72 ± 8.16	0.196 ± 0.100	− 27.74 ± 8.50
CDSC_6_	206.09 ± 22.09	0.122 ± 0.014	− 29.61 ± 2.06

All data are expressed as mean ± SD of three independent experiments

**Fig. 2:**
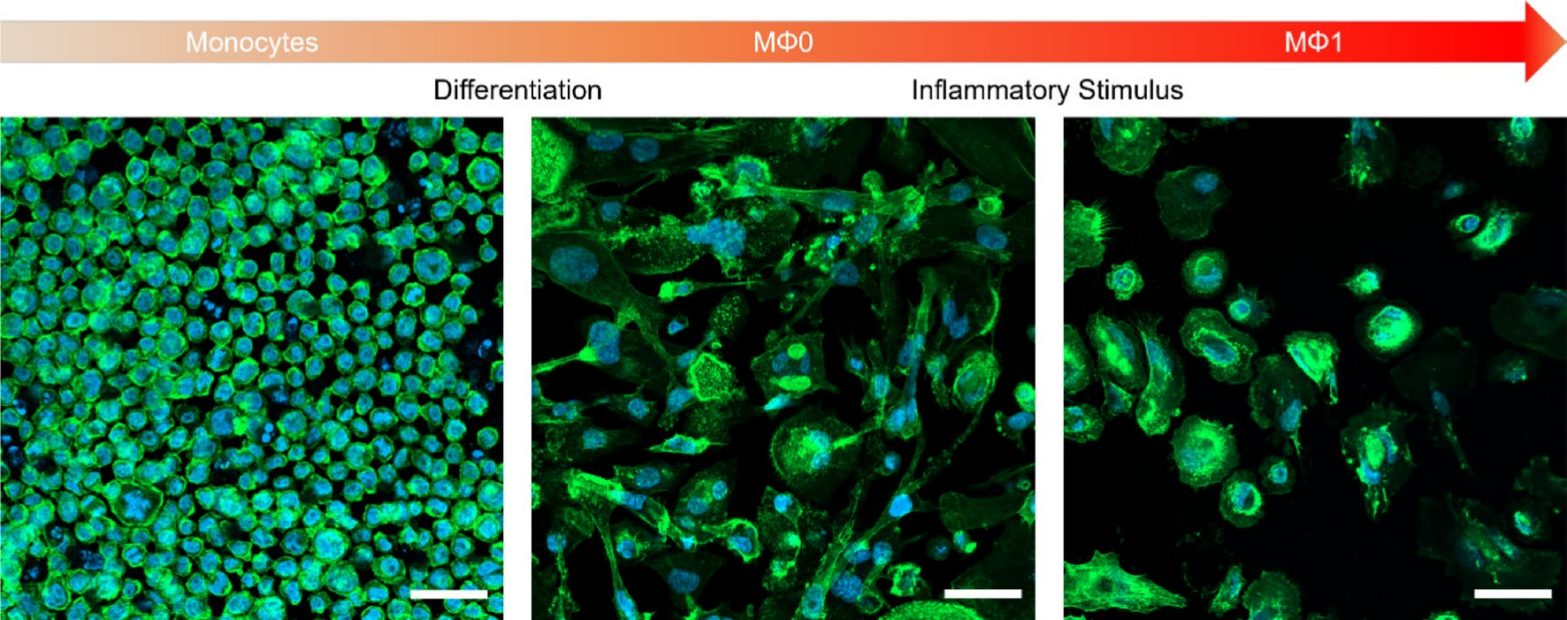
Confocal laser scanning micrographs of native monocytes (left) differentiated to MΦ0 macrophages via PMA addition (middle). Resting macrophages were further polarized to MΦ1 cells via LPS and IFNγ supplementation (right). Actin (green, Alexa Fluor 488) and cell nuclei (blue, DAPI) were visualized via immunocytochemistry. Scale bars depict 50 µm

Due to their involvement in all stages of inflammation, macrophages are favored targets for various immunomodulatory therapy approaches. To evaluate the intrinsic effect of the CD-NPs on human macrophages, we differentiated respective monocytes into resting MΦ0 macrophages. This transition is characterized by an increase in size and granularity as well as an elongated morphology. By adding classical pro-inflammatory stimuli (LPS and IFNγ), MΦ0 macrophages were polarized to activated MΦ1 macrophages, characteristic of the inflamed microenvironment [16, 39]. The resulting MΦ1 cells appeared to be more circular with a comparable granularity to MΦ0, indicating successful polarization of the cells (Fig. 2).

### Biocompatibility of cyclodextrin nanoparticles

**Fig. 3:**
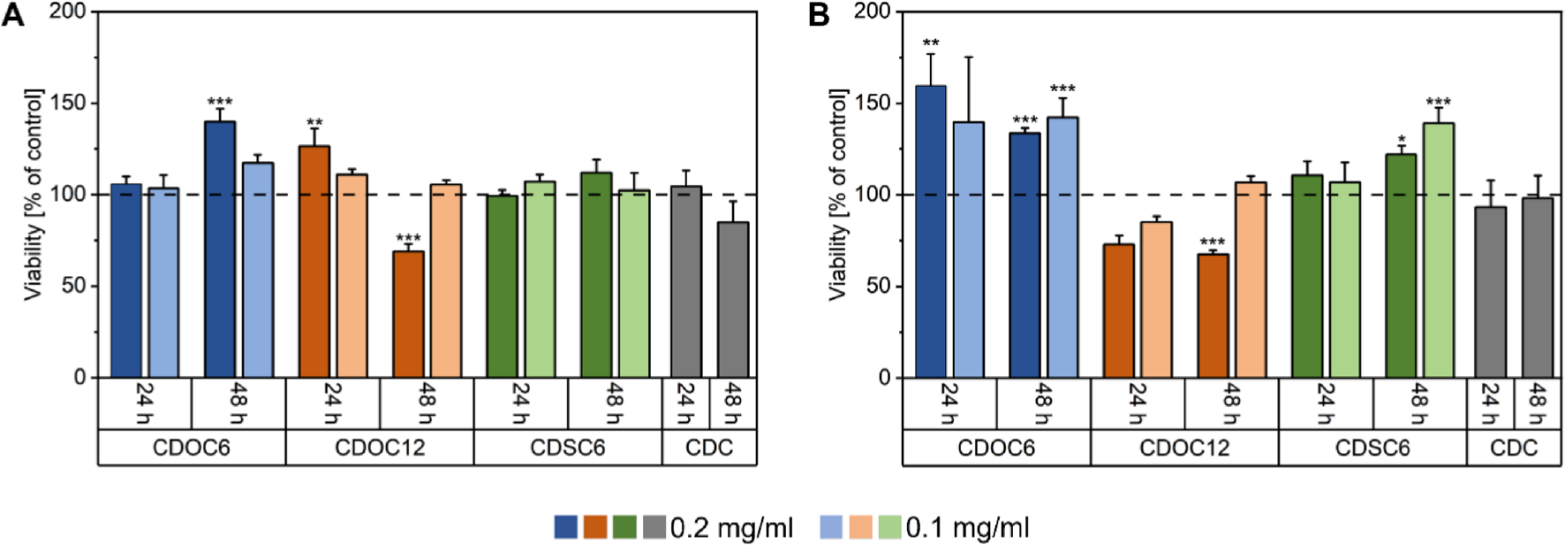
Viability of **A** MΦ0 and **B** MΦ1 cells after 24 h and 48 h incubation with two different concentrations of nanocarriers, β-CD solution served as control (CDC). The concentrations refer to the applied nanocarrier mass. All data are expressed as mean ± SD of three independent experiments. Statistical analysis was performed by One-Way ANOVA with Dunn-Sidak posthoc test. *p < 0.05, **p < 0.01, and ***p < 0.001

To assess the biocompatibility of the nanocarriers, CD-NPs in two concentrations (0.2 mg ml^−1^, 0.1 mg ml^−1^) were applied to either MΦ0 or MΦ1; native β-CD in solution (CDC) served as control (Fig. 3A, B). Concerning MΦ0, no significant decrease in cell viability was observed after 24 h incubation with CDOC_6_- or CDSC_6_-NP. However, resting macrophages treated with 0.2 mg ml^−1^ CDOC_12_-NPs exhibited a significant increase in cell viability compared to the control. Interestingly, 48 h supplementation with 0.2 mg ml^−1^ CDOC_6_-NPs led to a noticeable improvement in cell viability, whereas incubation with the highest concentration of CDOC_12_-NPs resulted in a sharp decline. The lower concentrations of both CD-NPs did not alter the viability compared to the untreated control. CDSC_6_-NPs and β-CD supplementation did not significantly affect MΦ0 vitality in any concentration tested here (Fig. 3A). In the case of MΦ1 cells, incubation with CDOC_6_-NPs led to a strong incline in cell viability regarding both concentrations and time points, which was also highly significant except for the 0.1 mg ml^−1^ CD-NP treatment at 24 h. The viability of the pro-inflammatory macrophages was also enhanced after treatment with both concentrations of CDSC_6_-NPs after 48 h. Regarding treatment with CDOC_12_-NPs, the same concentration-dependent cellular toxicity measured for MΦ0 was visible for MΦ1 after 48 h. β-CD supplementation did not affect MΦ1 cell viability at both time points analyzed (Fig. 3B).

### Regulatory effect of CD-NPs on gene expression

**Fig. 4:**
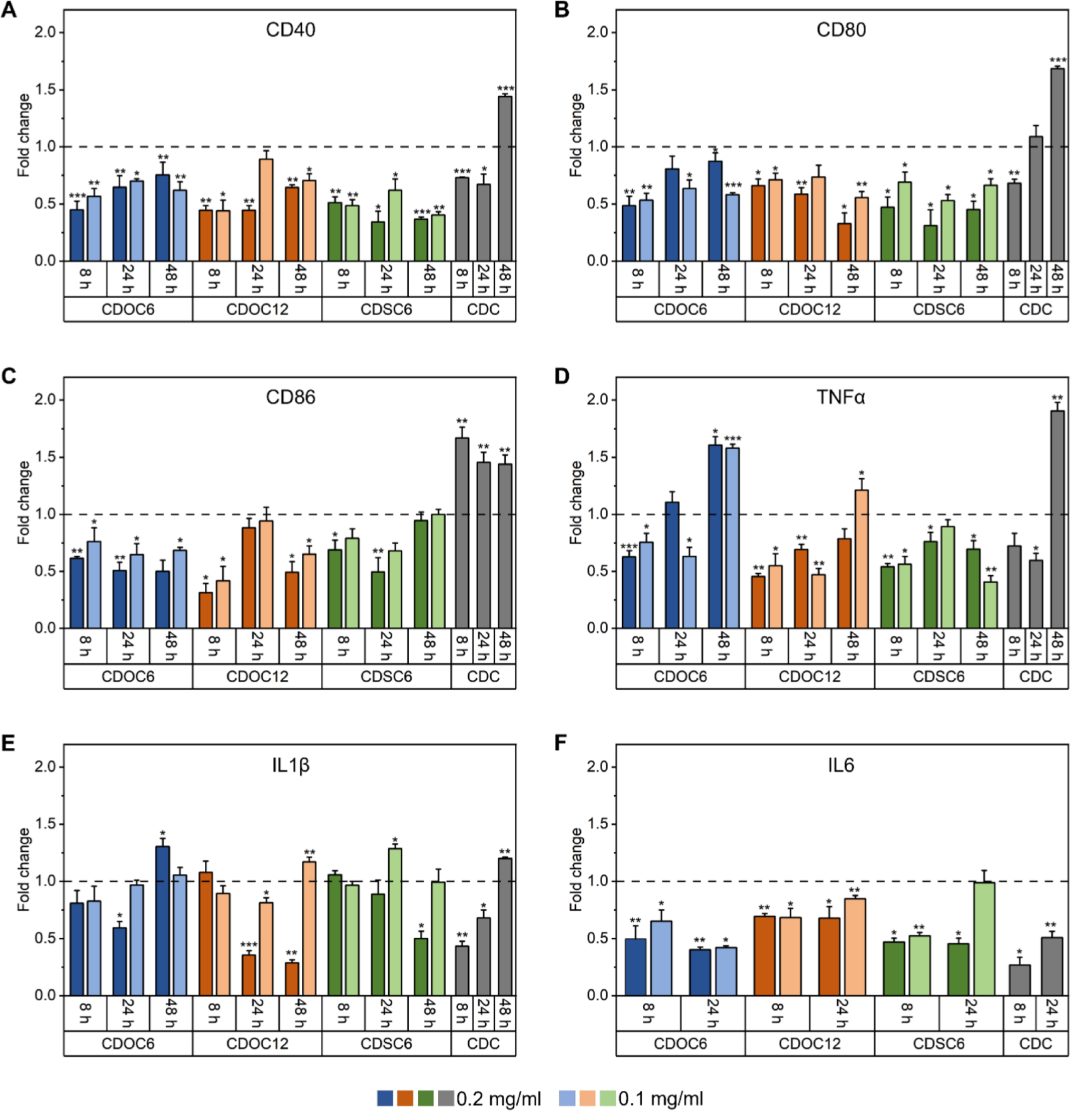
mRNA level of **A** CD40, **B** CD80, **C** CD86, **D** TNFα, **E** IL1β, and **D** IL6 measured via RT-qPCR. Data are displayed as fold change against the untreated MΦ1 control and are depicted as mean ± SD of three independent experiments. Statistical analysis was carried out via One-Way ANOVA with Dunn–Sidak posthoc test or unpaired t-test if no multiple comparisons against the same control were performed. *p < 0.05, **p < 0.01, and ***p < 0.001

First, the anti-inflammatory properties of CD-NPs were evaluated via RT-qPCR. Therefore, messenger RNA (mRNA) levels of three MΦ1-specific surface markers (CD40, CD80, and CD86) and three pro-inflammatory cytokines (TNFα, IL1β, and IL6) were analyzed after treatment with CD-NPs in two different concentrations (0.2 and 0.1 mg ml^−1^) for 8 h, 24 h, and 48 h. β-CD solution at 0.2 mg ml^−1^ was used as a control (CDC), and all measurements are expressed as fold change against a medium-treated MΦ1 control. CD40 mRNA levels were significantly and concentration-dependently decreased for all time points after treatment with all CD-NP types; native β-CD solution decreased CD40 mRNA abundance after 8 h and 24 h, whereas transcription increased after 48 h (Fig. 4A). Regarding CD80, all particle types decreased the mRNA level with minor exceptions for CDOC_6_ at 0.2 mg ml^−1^ and CDOC_12_ at 0.1 mg ml^−1^. The β-CD control affected the CD80 mRNA level similarly to CD40 (Fig. 4B). In contrast, mRNA levels of the surface marker CD86 were increased after β-CD treatment. CDOC_6_-NPs application significantly lowered the expression of this surface marker over all analyzed time points and concentrations. Adding CDOC_12_- and CDSC_6_-NPs to the MΦ1 cells decreased the expression of CD86 with minor exceptions at 24 h and 48 h regarding the ester and thioether derivatives, respectively (Fig. 4C).

In contrast to the overall reducing effects of CDOC_6_-NPs on surface marker expression, both concentrations of this derivative only lowered the mRNA level of TNFα at 8 h; a similar decrease was visible for the lower concentration at 24 h. Interestingly, 48 h treatment with CDOC_6_-NPs resulted in a significant increase in intracellular TNFα mRNA level. The same pattern was observed for native β-CD and CDOC_12_-NPs but with a significantly lower mRNA level at 24 h after treatment with the latter. CDSC_6_ nanocarriers, in contrast, induced the highest reduction of TNFα coding mRNA, decreasing its level over all time points (Fig. 4D). Comparing the mRNA level of all cytokines, IL1β showed the least susceptibility to the treatment with the CD-NPs. A decrease in IL1β-coding mRNA was only measured for CDOC_6_-NPs at 24 h, and CDSC_6_-NPs at 48 h after application of 0.2 mg ml^−1^, respectively. CDOC_6_-based CD-NPs even had a slightly pro-inflammatory effect after 48 h, indicated by the increase in IL1β mRNA. Incubation of MΦ1 cells with CDOC_12_-NPs resulted in the most pronounced reduction of IL1β mRNA, significantly lowering the level after 24 h and 48 h. In contrast, incubation with the soluble β-CD decreased the mRNA level after 8 h and 24 h incubation but led to an increase after 48 h (Fig. 4E). IL6 mRNA levels could only be measured after 8 h and 24 h cultivation and were not detectable after 48 h. IL6 mRNA showed the highest susceptibility to CD-NP treatment with reduced levels at all time points and all concentrations, with a minor exception for CDSC_6_-NPs after 24 h at 0.1 mg ml^−1^. β-CD treatment also lowered IL6 expression compared to the untreated MΦ1 control (Fig. 4F).

### Determination of pro-inflammatory cytokine secretion

**Fig. 5:**
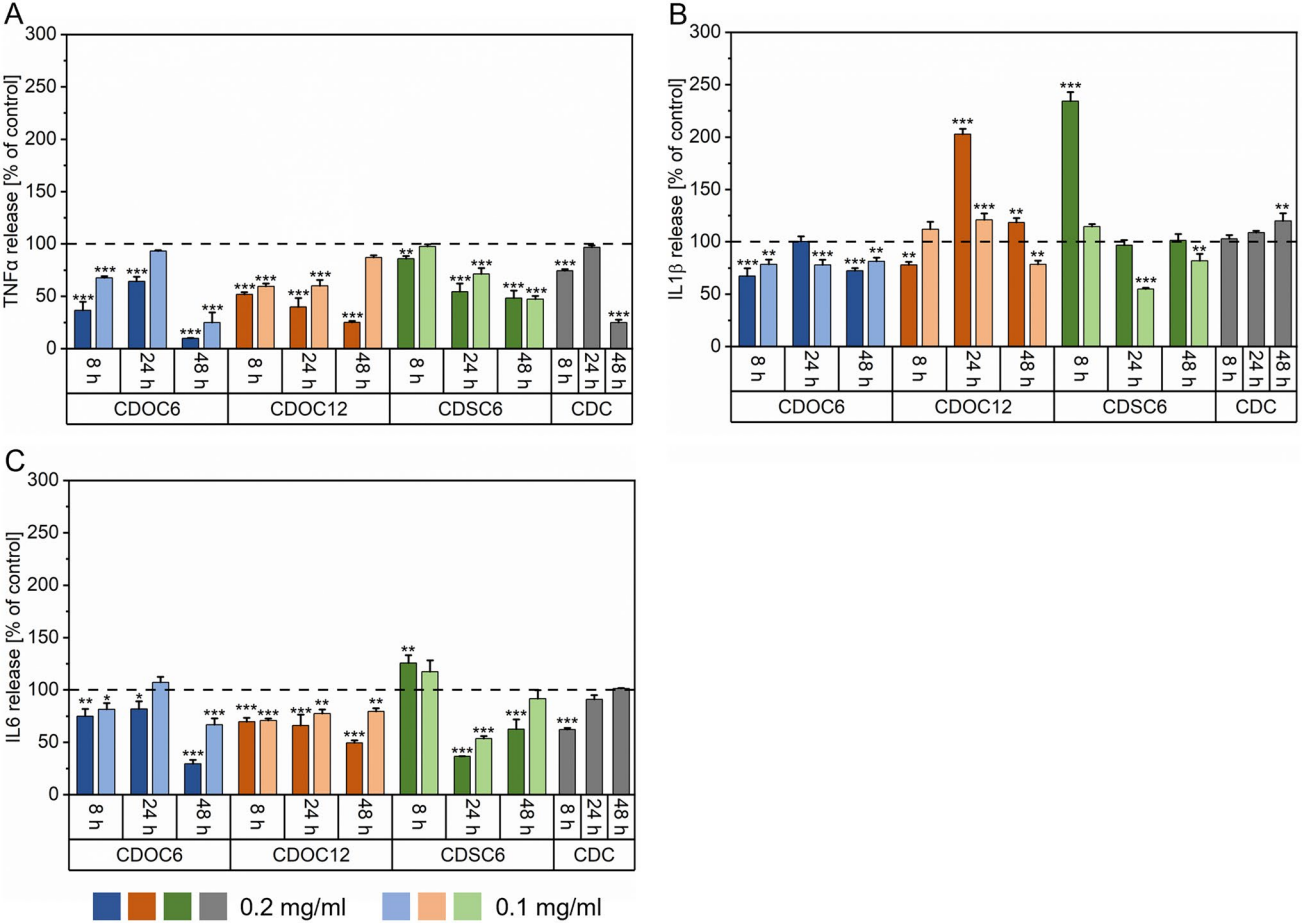
**A** TNFα, **B** IL1β, and **C** IL6 secretion of MΦ1 after incubation with CD-NPs or CDC at 8 h, 24 h, and 48 h quantified via ELISA. All data were compared to an untreated MΦ1 control and are expressed as mean ± SD of three independent experiments. Statistical analysis of cytokine release was carried out via One-Way ANOVA with Dunn-Sidak posthoc test. *p < 0.05, **p < 0.01, and ***p < 0.001

In addition to the evaluation of mRNA level, the anti-inflammatory potential of CD-NPs was quantified by measuring secreted TNFα, IL1β, and IL6 using supernatant of untreated MΦ1 cells as control. CDOC_6_-NP treatment significantly lowered TNFα secretion and was dependent on the concentration for all time points with a maximum effect after 48 h. CDOC_12_-NPs addition resulted in a comparable TNFα reduction, while the most substantial effect was observed 48 h after application of 0.2 mg ml^−1^. Although CDSC_6_-NPs supplementation resulted in the lowest reduction of TNFα secretion compared to the other CD-NPs, the effect was sustained over all sampling points for all concentrations, culminating in a substantial diminution of the cytokine after 48 h. Soluble β-CD reduced the TNFα release after 8 h and 48 h incubation, comparable to CDSC_6_-NPs, but showed no significant effect after 24 h. (Fig. 5A). In the case of IL1β, addition of CDOC_6_-NPs lowered its secretion over all analyzed time points. CDOC_12_-NPs only decreased the cytokine release at 8 h, whereas a significant induction of IL1β secretion was measured after 24 h and 48 h incubation for 0.2 mg ml^−1^ CDOC_12_-NPs. In contrast, CDSC_6_-based NPs heavily increased IL1β secretion at 8 h in a strong concentration-dependent manner. This pro-inflammatory effect was attenuated, and significantly lower extracellular IL1β levels were measured for samples treated with 0.1 mg ml^−1^ CDSC_6_-NPs. β-CD addition resulted in no alterations after 8 h and 24 h but a slight increase in IL1β secretion after 48 h (Fig. 5B). Concerning IL6 secretion, CDOC_6_- and CDOC_12_-NPs significantly reduced the secretion of this pro-inflammatory cytokine over all sample points, with the highest decrease after 48 h; a concentration dependency was primarily apparent for the CDOC_6_ treatment. Although the application of CDSC_6_-NPs led to a slight increase in the release of IL6, its secretion was notably reduced after 24 h and 48 h compared to the untreated MΦ1 control. The native β-CD control significantly lowered extracellular IL6 after 8 h, but the addition of this soluble CD had no measurable effect after 24 h and 48 h. (Fig. 5C).

### Subcellular localization of the inflammasome

**Fig. 6:**
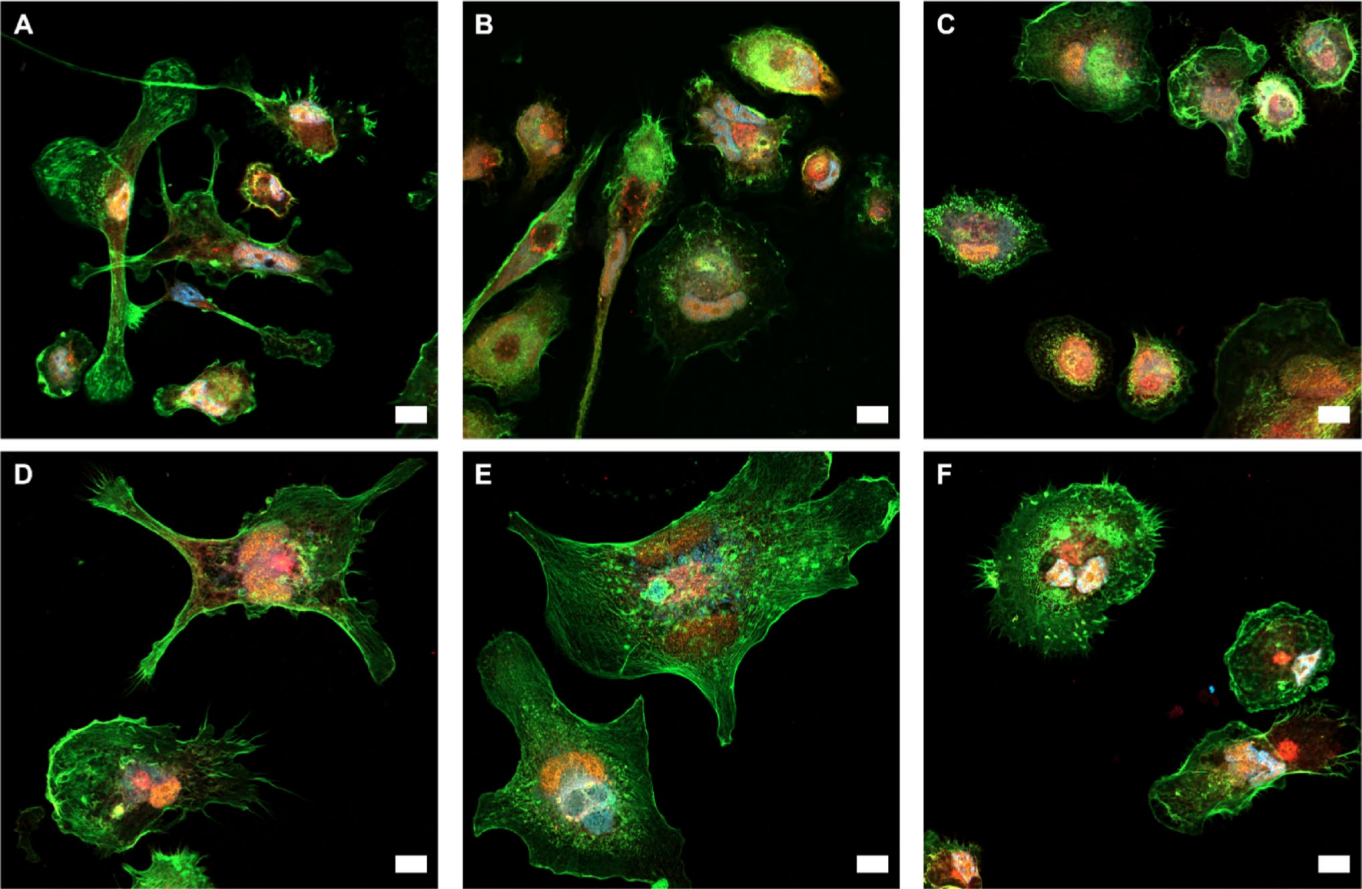
Confocal laser scanning micrographs of **A** MΦ0, **B** MΦ1 and MΦ1 cells incubated with 0.2 mg ml^−1^ of **C** native β-CD or nanocarriers prepared from **D** CDOC_6_, **E** CDOC_12_, and **F** CDSC_6_, visualized by staining of NLRP3 (red, Alexa Fluor 633), ASC (yellow, Alexa Fluor 555), actin (green, Alexa Fluor 488), and nuclei (blue, DAPI). Scale bars depict 10 µm

To evaluate the influence of native β-CD and the amphiphilic CD nanocarriers on the inflammasome, the cellular distribution of two key inflammasomal proteins, NLRP3 (NLR family pyrin domain containing 3) and ASC (apoptosis-associated speck-like protein containing a CARD), was qualitatively analyzed in particle-treated macrophages using confocal laser scanning microscopy. Concerning MΦ0 macrophages, NLRP3 was primarily found in the cytoplasm, whereas ASC was highly co-localized with the cell nucleus (Fig. 6A). In MΦ1 cells, both proteins are inversely localized with NLRP3 at the nucleus and ASC in the cytoplasm (Fig. 6B). Visualization of ASC in stimulated macrophages after treatment with β-CD showed a robust co-localization with the macrophage nuclei. However, NLRP3 was also perinuclearly localized (Fig. 6C). Treating MΦ1 cells with 0.2 mg ml^−1^ CDOC_6_-NPs led to a shift of NLRP3 to the cytoplasm, whereas ASC could be found highly co-localized with the DAPI signals (Fig. 6D). A similar distributional pattern could be visualized for CDOC_12_ treatment, showing a broader distribution of NLRP3 signals in the cell and a perinuclear appearance of ASC (Fig. 6E). Regarding CDSC_6_, NLRP3-localization showed a similar redistribution, whereas the effect on ASC was not as pronounced as the other amphiphilic CD types (Fig. 6F).

**Fig. 7:**
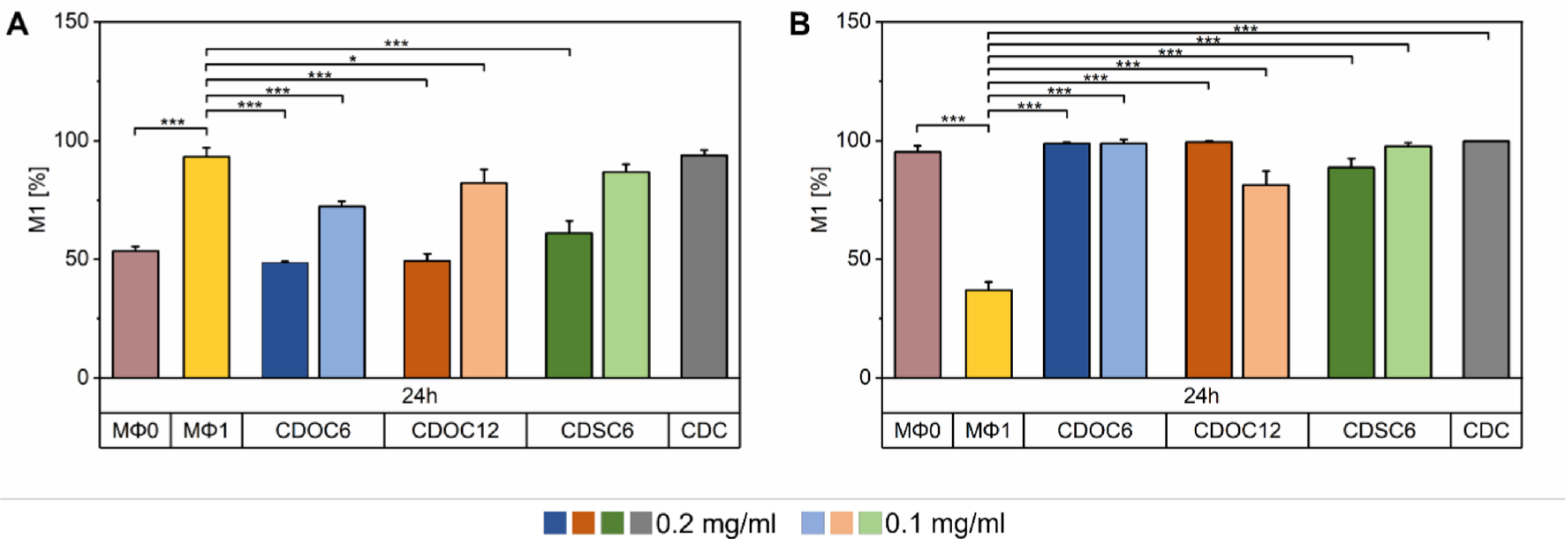
Manders' coefficient 1 evaluation of **A** NLRP3 and **B** ASC calculated from micrographs depicting treated or untreated macrophages after immunofluorescent staining (Online Resources S1 and S2). All data are expressed as mean ± SD of three independent experiments. Statistical analysis was carried out via One-Way ANOVA with Dunn-Sidak posthoc test. *p < 0.05, **p < 0.01, and ***p < 0.001

The co-localization of NLRP3 and ASC was quantitatively assessed via Mander's coefficient 1 (M1) calculated out of three representative images for the MΦ0 and MΦ1 control, each CD-NP type in both concentrations, and the β-CD treatment (Fig. 7). M1 coefficients express the total pixel intensities of either NLRP3 or ASC fluorescence signals that co-localize with the nucleus-correlating DAPI signals in relation to the total intensity of NLRP3 or ASC. For this computation, correlative laser scanning microscopy micrographs of treated cells and the respective controls were analyzed using the ImageJ Plugin JACoP (see Online Resources S1 and S2). Regarding NLRP3, cytoplasmatic distribution in MΦ0 cells could be confirmed via M1 coefficient, significantly differing from the nuclearly localized NLRP3 in MΦ1. Adding CDOC_6_-, CDOC_12_-_,_ and CDSC_6_-NPs notably enhanced the cytoplasmatic localization of NLRP3 in a concentration-dependent manner. The treatment with 0.1 mg ml^−1^ CDSC6-NPs was the exception, showing no significant change compared with MΦ1-localized NLRP3. Addition of β-CD also resulted in no alteration of the NLRP3 distribution (Fig. 7A). The subcellular localization of ASC was similarly quantified. The perinuclear occurrence of the protein in resting macrophages, as well as the broad distribution of ASC in MΦ1, was confirmed. The different nanoparticle types and the β-CD solution concentration independently induced the re-localization of MΦ1 ASC towards the perinuclear space (Fig. 7B).

### Change of lipidomic profile

**Fig. 8:**
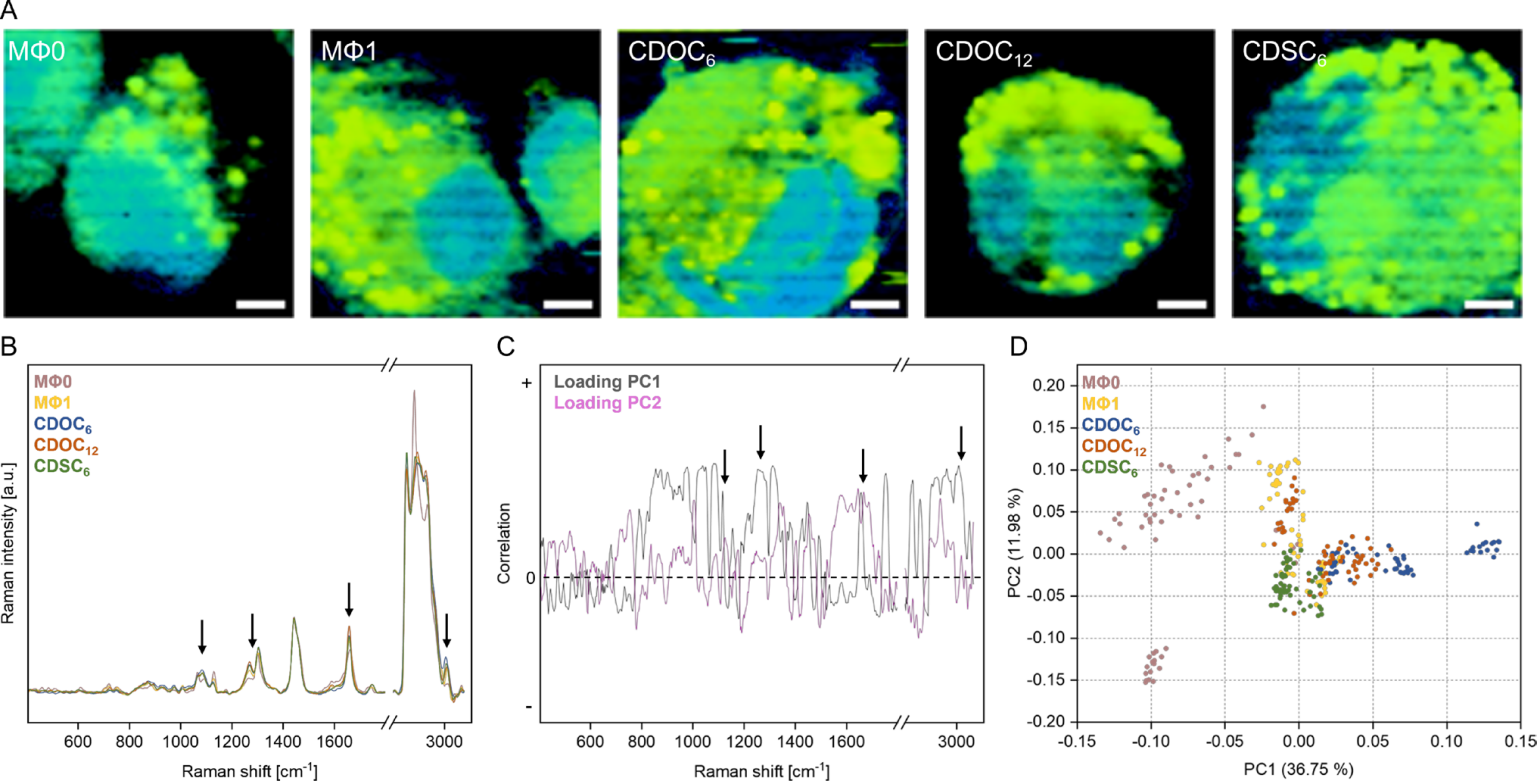
**A** Raman false-color images of MΦ0, MΦ1, and MΦ1 treated with CDOC_6_, CDOC_12_, and CDSC_6_ visualized by nuclei (blue) and lipid droplets (yellow). Scale bars depict 5 µm. **B** Raman spectra of untreated MΦ0(brown) and MΦ1 (yellow) cells and of MΦ1 treated with CDOC_6_ (blue), CDOC_12_ (red), and CDSC_6_ (green). **C** Loadings of the first two principal components: PC1 (black) and PC 2 (purple) of the lipid spectra. **D** Score plot of the first two principal components (PC1 and PC2) of the obtained Raman spectra

Confocal Raman imaging was used to evaluate the intracellular lipid distribution and composition of CD-NP-treated MΦ1 cells. Images of untreated and particle-treated MΦ1 macrophages revealed a tendency to higher amounts of lipid droplets than the untreated MΦ0 control (Fig. 8A). The Raman spectrum at 2883 cm^−1^ had the highest intensity for MΦ0, whereas the intensity of the signals at 1085–1086, 1120–1129, 1260–1269, 1656, 1658, 1670, and 3000–3010 cm^−1^ was higher compared to untreated and treated MΦ1. In addition, these signals showed high variation regarding their intensities after treatment with CD-NPs and were marked for further evaluation (Fig. 8B). Principal component analysis was performed to reduce the Raman spectra's dimensionality and differentiate the spectral information of the different cell samples. At first, principal component 1 (PC1) and principal component 2 (PC2) loadings were calculated, guiding the interpretation of the principal component analysis. The loading of the first two principal components accounted for 36.75% and 11.98% of the variance explained, respectively (Fig. 8C). Positively correlated peaks with each principal component had values above 0 in the loading plot, and negatively correlated peaks vice versa. The same signals in the Raman spectra were marked in the PC loading plot. The score plot of PC1 and PC2 showed the populations of MΦ0, MΦ1, and particle-treated MΦ1 cells. PC1 differentiated the groups, whereas PC2 accounted for less variation between the groups but seemed to cover intragroup variability (Fig. 8D). Signals obtained by scanning resting macrophages clustered mainly on the left side of the PCA plot. In contrast, the polarization into MΦ1 and the CD-NP treatment led to a right shift of the correlating clustered signals. Especially the clusters of CDOC_6_-NPs showed a higher PC1 variability compared to all other treatments and controls. Signals of CDSC_6_-NPs-treated MΦ1 cells showed a comparatively low group variability (PC2). However, they mainly clustered together with the untreated MΦ1 population. In contrast, PC2-dependent variability was higher for cells after CDOC_12_-NP treatment, but clustering revealed a higher variance compared to the MΦ1 control.

## Discussion

Inflammation is a highly controlled biological response to harmful stimuli involving various cell types and intricate signaling cascades. Dysregulated resolution of inflammatory processes, resulting in prolonged or chronic inflammation, cumulates in severe tissue damage and is a significant challenge for the therapy of immune-mediated disorders. Especially concerning the high complexity and heterogeneity of inflammatory responses, biomaterial-based strategies hold great potential based on their adaptability. A relatively novel class of biomolecules with intrinsic immunomodulatory properties are CDs, cyclic oligosaccharides based on glucose monomers. By altering the chain length and linking structure of classic β-CDs, derivatives with distinct physicochemical properties were synthesized, enabling the formation of supramolecular and subsequent particulate or vesicular structures depending on the chemical modification [36]. These CD-based nanoparticles were applied to pro-inflammatorily-primed macrophages to analyze and evaluate their intrinsic immunomodulating potential compared to soluble β-CD. After analyzing biocompatibility, mRNA levels, cytokine release, and potential effects on the localization of inflammasomal proteins were assessed. Furthermore, their influence on the lipidomic profile of MΦ1 cells was investigated via Raman microscopy.

The initial assessment of physicochemical characteristics revealed distinct features for each CD-NP type. The higher lipophilicity of the thioether linkage in CDSC_6_, compared to the ester linkage in other variants, enhanced molecular attraction, resulting in larger particles over 200 nm in diameter. The angled ester bond in CDOC_6_ and CDOC_12_ sterically hindered molecule accumulation more than the linear thioether. Among ester-bonded derivatives, CDOC_12_ formed larger particles due to its longer carbon chain. Notably, CDOC_12_- and CDSC_6_-NPs had similar particle sizes, highlighting the significant influence of linking structure over aliphatic chain length. The increased polydispersity of CDOC_12_-NPs by nearly 0.2 is attributed to its longer carbon chain, leading to more random assembly and broader particle distribution. Despite the variations, all particle sizes and dispersity remained within the nanometer range, enabling uptake via phagocytosis or clathrin-dependent endocytosis by macrophages [36, 40–42]. Additionally, soluble CDs are known for their cholesterol depletion ability, presenting a possible interaction mechanism with the cell membrane of macrophages in addition to common endocytotic mechanisms [43–45].

Although native CDs show no specific toxicity and previous studies found no significant cytotoxicity of CD-based NPs in undifferentiated THP-1 cells, we further assessed the biocompatibility of CD-NPs in differentiated and stressed phagocytic cells [29, 36]. After applying CDOC_6_-NPs, a notable increase in viability was observed in in resting MΦ0 and pro-inflammatory MΦ1 cells, which was not replicated by the β-CD control, suggesting no attribution to the CD-backbone. We analyzed cell viability changes in resting MΦ0 and pro-inflammatory MΦ1 cells. While studies on caproic acid, the fatty acid attached to the CD-backbone of CDOC_6_ are rather ambiguous, such as enhancing keratinocyte viability but being cytotoxic to various cancer cells, we attributed the increased viability to intrinsic, particle-specific effects [46–48]. CDSC_6_-NPs also improved cell viability, albeit slightly less. In case of CDOC_12_-NPs, a concentration-dependent cytotoxicity after treatment for 48 h was detected that might be correlated to the lauric acid attached to the CD-core in these nanoparticles, which is known for its adverse cellular effects, especially concerning macrophages. Thus, the observed decrease in viability might result from the increased uptake by the phagocytotic cells and the subsequent degradation of the particles, leading to intracellular disaffiliation of the fatty acid [49–51]. Overall, amphiphilic CD NPs demonstrate promising potential to boost cell viability, depending on their concentration and physicochemical properties, by relieving cell stress, enhancing metabolism, or interfering with apoptosis.

The potential anti-inflammatory properties of the three different nanoparticle formulations were further assessed on mRNA and protein levels using classic pro-inflammatory MΦ1 markers. After applying all particle types, we observed a significant downregulation of mRNA transcription for the surface markers CD40, CD80, and CD86. This drastic effect could not be entirely reproduced by the β-CD control, as the expression of surface markers increased again after 48 h treatment, pointing to backbone-independent mechanisms of the CD-NPs. Interestingly, the fatty acids grafted onto the CD-backbone are known to increase the expression of the analyzed surface markers, indicating that mRNA alterations are probably not associated with the grafted aliphatic chains [52, 53]. This more pronounced and prolonged effect of the CD-NPs on surface marker mRNA levels compared to native β-CD indicated a specific particle-mediated mechanism that should be further evaluated. As the expression of the analyzed surface markers is stimulated by IFNγ, adsorption of this glycoprotein onto the nanoparticles might be possible [54–56]. However, any mechanism mediated by CD-NP application resulting in alterations of IFNγ levels could be a promising start leading to treatment options for various cancer types, autoimmune diseases, chronic inflammation, and even allergic conditions that are associated with high levels of CD40, CD80, and CD86 [57–65].

We also evaluated changes in mRNA levels and protein secretion of TNFα, IL1β, and IL6, three key pro-inflammatory cytokines involved in acute and chronic inflammation. These cytokines trigger various molecular pathways, causing fever, cyclooxygenase activation, and cell death [66–69]. TNFα, in particular, is a major target for pharmacological treatments, with biologics reducing its extracellular protein levels significantly improving patient care [70–72]. CDOC_6_-, CDOC_12_-, and CDSC_6_-NPs lowered the expression of all pro-inflammatory cytokines studied. However, the reduction in cytokine mRNA levels was more variable than the downregulation of surface markers. For instance, CDOC_6_-NPs initially reduced TNFα mRNA but later caused an increase, similar to the effect observed with the native β-CD control, suggesting an influence of the CD-backbone and contrasting literature on caproic acid, which typically decreases TNFα mRNA [73]. The initial TNFα mRNA decrease was sustained by CDOC_12_- and CDSC_6_-NPs, potentially due to a combined effect of the CD-backbone and the particulate structure. Regarding IL1β, the immediate anti-inflammatory effect seen with native β-CD at 8 and 24 h was delayed with CDOC_12_- and CDSC_6_-NPs, which showed significant mRNA reductions only after 24 and 48 h, respectively. Like TNFα, CDOC_6_-NPs and β-CD maintained an anti-inflammatory effect for up to 24 h but not beyond. The significant decrease in IL6 mRNA levels observed with all particle types likely stems from the CD-backbone, as similar fold changes were noted with the native β-CD solution. Treatment with CDOC_12_- and CDSC_6_-NPs significantly reduced extracellular TNFα levels, consistent with the observed mRNA changes. In contrast, CDOC_6_-NPs minimized TNFα secretion after 48 h, despite increased mRNA levels at that time, likely due to a delay between mRNA elevation, protein translation, and TNFα secretion. The similarity between CDOC_6_-NPs and native β-CD in TNFα release suggests a CD-backbone-mediated mechanism. IL1β secretion, independent of the CD-backbone, decreased with the application of CDOC_6_-NPs, while treatment with CDSC_6_- and especially CDOC_12_-NPs increased extracellular IL1β, likely due to lauric acid in CDOC_12_, which is known to enhance IL1β release [74]. The lack of correlation between IL1β mRNA and extracellular cytokine levels suggests complex downstream processes, warranting further investigation into intracellular protein levels for insights into post-transcriptional or translational regulation. In contrast, IL6 release mirrored mRNA downregulation, particularly with CDSC_6_-NP treatment, where mRNA decline consistently preceded reduced protein levels. Beyond the direct mechanistic effects of CD-based particles or their degradation products, CD-NPs may also bind extracellular pro-inflammatory proteins. This scavenging effect, reported for other polysaccharide-based biomaterials with anti-inflammatory properties, could explain the significant reduction in extracellular cytokines and differences in particle performance [9, 36, 75, 76]. For instance, direct adsorption of pro-inflammatory stimuli, such as LPS or IFNγ, onto the surface of CDOC_6_- and CDSC_6_-NPs could be favorable, while the large aliphatic chains of CDOC_12_ might negatively influence or ultimately hinder such adsorbent effects [77, 78].

We further investigated changes in the pro-inflammatory phenotype through immunocytochemical analysis of the inflammasome, a key regulator of inflammation and innate immune responses. This multi-protein complex, composed of sensor molecules, adapter proteins, and effector caspases, detects pathogen- and danger-associated molecular patterns, triggering cytokine production and pyroptotic cell death [79–81]. We labeled NLRP3 and ASC, two main components, to determine their cellular distribution. In resting MΦ0 macrophages, NLRP3 is localized in the cytoplasm, and re-locates towards the nucleus upon pro-inflammatory stimuli, where it detects damaged endocytosed cells and increases cytokine release [82–84]. Furthermore, NLRP3 activation is associated with atherosclerosis, neuroinflammation, gout, and hemorrhagic stroke [85–89]. In contrast, ASC, the critical adapter protein for inflammasome activation, is perinuclearly located in resting macrophages and spreads during their activation to MΦ1 [90–92]. Fluorescence imaging confirmed the cellular localization of both proteins and Manders' coefficient 1 was used to quantify their co-localization with cell nuclei. After CD-NP treatment, NLRP3 relocated to the cytoplasm in a concentration-dependent manner, while ASC returned to its perinuclear location. This effect on NLRP3 seemed intrinsic to CD-NPs, as β-CD showed no significant impact. However, β-CD strongly influenced ASC's perinuclear relocation, suggesting the CD-backbone might mediate this effect after CD-NP treatment.

The anti-inflammatory effects of CD NPs often mirrored those of native β-CD, indicating that the CD-backbone may partially convey immunoregulatory effects through the modulation of the Liver X receptor (LXR) pathway [17]. This signaling cascade is crucial for macrophages in preventing excessive autoimmune reactions due to increased cholesterol intake after digestion of endogenous cells. Cholesterol and its derivatives, like oxysterol, act as agonists for the nuclear LXR(α) receptor, repressing the expression of several cytokines like TNFα, IL1β, and IL6 in macrophages [93, 94]. Soluble CDs enhance the solubility of cholesterol crystals, increasing LXR(α) agonists and activating the LXR pathway, even without cholesterol crystals present [17]. Reduced cytokine expression and protein levels observed after CD-NP addition may be attributed to LXR pathway activation by the particles or their degradation products acting as intrinsic agonists. Intracellular cholesterol levels may further explain the differing effects of β-CD and CD NPs on NLRP3. While cholesterol activates NLRP3, encapsulation or adsorption of cholesterol by CD NPs—but not β-CD—could downregulate NLRP3 activity, leading to its relocation [44, 45, 95, 96]. The solid-state and chemical modifications of CD-NPs might be crucial for simultaneously activating the LXR pathway and deactivating NLRP3 by acting as intracellular cholesterol transport agents. Future experiments should address the complex regulation of the LXR pathway and the molecular mechanisms behind the anti-inflammatory properties of CD NPs.

Potential influences on the complete lipidomic profile of the macrophages were investigated using confocal Raman microscopy. This label-free technique was used to evaluate potential alterations of the lipid droplet composition of untreated and particle-treated pro-inflammatory macrophages. Previous studies showed that macrophage polarization leads to various changes in the cellular lipid metabolism, including an elevated level of triglycerides and cholesterol esters after Toll-like receptor 4 activation in pro-inflammatory-primed MΦ1 [97, 98]. Especially in macrophages, lipidomic remodeling seems to be an essential adaptation upon inflammatory stimulation, resulting in significant downstream effects in various cellular functions [98–100]. Accordingly, we found distinct differences in the amount and distribution of lipid droplets in MΦ0 and MΦ1 cells. After incubation of MΦ1 with CD-NPs, the amount of unsaturated fatty acids was enhanced measurably by increased signal intensities of gauche C–C stretching (1085–1086 cm^−1^), =CH deformation (1260–1269 cm^−1^), C=C stretching (1658 cm^−1^) and =CH stretching (3000–3010 cm^−1^). Furthermore, Raman signals of MΦ0 macrophages indicated a *trans* conformation of unsaturated fatty acids (1120–1129 cm^−1^ and 1670 cm^−1^), whereas a robust *cis* conformation signal was visible in untreated- and particle-treated MΦ1 cells (1656 cm^−1^) [101–103]. Additionally, these differences in the lipid composition were analyzed via principal component analysis. The signals mentioned above related to increased amounts of unsaturated fatty acids were positively correlated with PC1. Since CDOC_6_- and CDOC_12_-NP treatment showed the most significant increase of PC1 in the PCA score plot, CDOC_6_- and CDOC_12_-NPs had the most influence on the lipidomic alteration towards unsaturated fatty acids. Increasing oxidation of fatty acids, leading to a higher amount of intracellular unsaturated fatty acids, is a crucial mechanism of anti-inflammatory macrophages [99, 104–106]. These findings highlight that CD-NPs alter many different cellular functions and characteristics, including marker gene expression, cytokine release, inflammasomal localization, and the lipidomic profile, thus, mediating significant anti-inflammatory effects in macrophages.

## Conclusion

Multifunctional biomaterials leveraging inherent material characteristics are valuable for targeting various stages of inflammation. Our CD-NPs surpassed the described anti-inflammatory effect of native β-CD, emphasizing the importance of their solid particulate structure and chemical modifications for the anti-inflammatory potential. Treatment of macrophages with CD-NPs significantly decreased mRNA and protein levels of pro-inflammatory cytokines, depending on the CD derivative used as a nanoparticle building block. They further induced the re-localization of two inflammasomal key proteins, indicating the deactivation of this complex. The CD-NPs additionally mediated an increase in lipid oxidation, shifting the lipidomic profile of pro-inflammatory towards anti-inflammatory macrophages. This study highlights the multifaceted benefits of CD-NPs for combating inflammatory processes, including the modulation of mRNA, protein, inflammasome, and lipid levels. Given the growing challenges in treating acute and chronic inflammation-associated diseases, where existing therapies often fall short, these findings offer promising perspectives for developing innovative therapeutic strategies. To further explore the potential of CD-NPs as a multifunctional biomaterial, extended in vivo characterization will provide valuable insights into their clinical utility, paving the way for translational applications and unlocking the potential of a multifunctioning anti-inflammatory drug delivery system.

## Supplementary Information

Additional file 1: Supplementary Fig. 1: Confocal laser scanning micrographs of untreatedMΦ0,MΦ1 and MΦ1 cells incubated with 0.2 mg ml^−1^ ofnative β CD, or CD NPs prepared fromCDOC_6_,CDOC_12_, andCDSC_6_, visualized by staining of NLRP3, ASC, actin, and nuclei. Scale bars depict 20 µm.

Additional file 2: Supplementary Fig. 2: Confocal laser scanning micrographs of MΦ1 cells incubated with 0.1 mg ml^−1^ nanocarriers prepared fromCDOC_6_,CDOC_12_,CDSC_6_, visualized by NLRP3, ASC, actin, and nucleusstaining. Scale bars depict 20 µm.

## Data Availability

The datasets used and/or analyzed in this study will be made available by the corresponding author upon reasonable request.

## Abbreviations

ASC: Apoptosis‑associated speck‑like protein containing a CARD
CD: Cyclodextrin
CDC: Native β-CD control
cDNA: Complementary deoxyribonucleic acid
CD-NPs: Amphiphilic cyclodextrin nanoparticles
CDOC_6_: Heptakis-6-O-hexanoyl-β-cyclodextrin
CDOC_12_: Heptakis-6-O-lauroyl-β-cyclodextrin
CDSC_6_: Heptakis-(6-deoxy-6-hexylthio)-β-cyclodextrin
DAPI: 4′,6-Diamidino-2-phenylindole
DLS: Dynamic light scattering
ELISA: Enzyme-linked immunosorbent assay
ELS: Electrophoretic light scattering
IFNγ: Interferon‑γ
IL: Interleukin
LPS: Lipopolysaccharides
LXR pathway: Liver-X-receptor pathway
M1: Manders' coefficient fraction 1
mRNA: Messenger ribonucleic acid
MTT: 3-(4,5-Dimethylthiazol-2-yl)-2,5-diphenyltetrazolium bromide assay
MΦ0: Resting macrophages
MΦ1: Activated, pro‑inflammatory macrophages
NLRP3: NLRP family pyrin domain containing 3
NP: Nanoparticle
PBS: Phosphate‑buffered saline
PC1/2: Principal component 1/2
PCA: Principal component analysis
PCR: Polymerase chain reaction
PDI: Polydispersity index
PMA: Phorbol‑12‑myristate‑13 acetate
RNA: Ribonucleic acid
RPMI: Roswell Park Memorial Institute 1640
RT: Room temperature
RT-qPCR: Real time quantitative PCR
SEM: Scanning electron microscopy
TNFα: Tumor necrosis factor α
