# Supplementary figures and images for: Anti-inflammatory effects of cyclodextrin nanoparticles enable macrophage repolarization and reduce inflammation
Source: Discov Nano. 2024 Dec 21;19(1):211. doi: 10.1186/s11671-024-04175-6 (PMC11662127; doi:10.1186/s11671-024-04175-6)

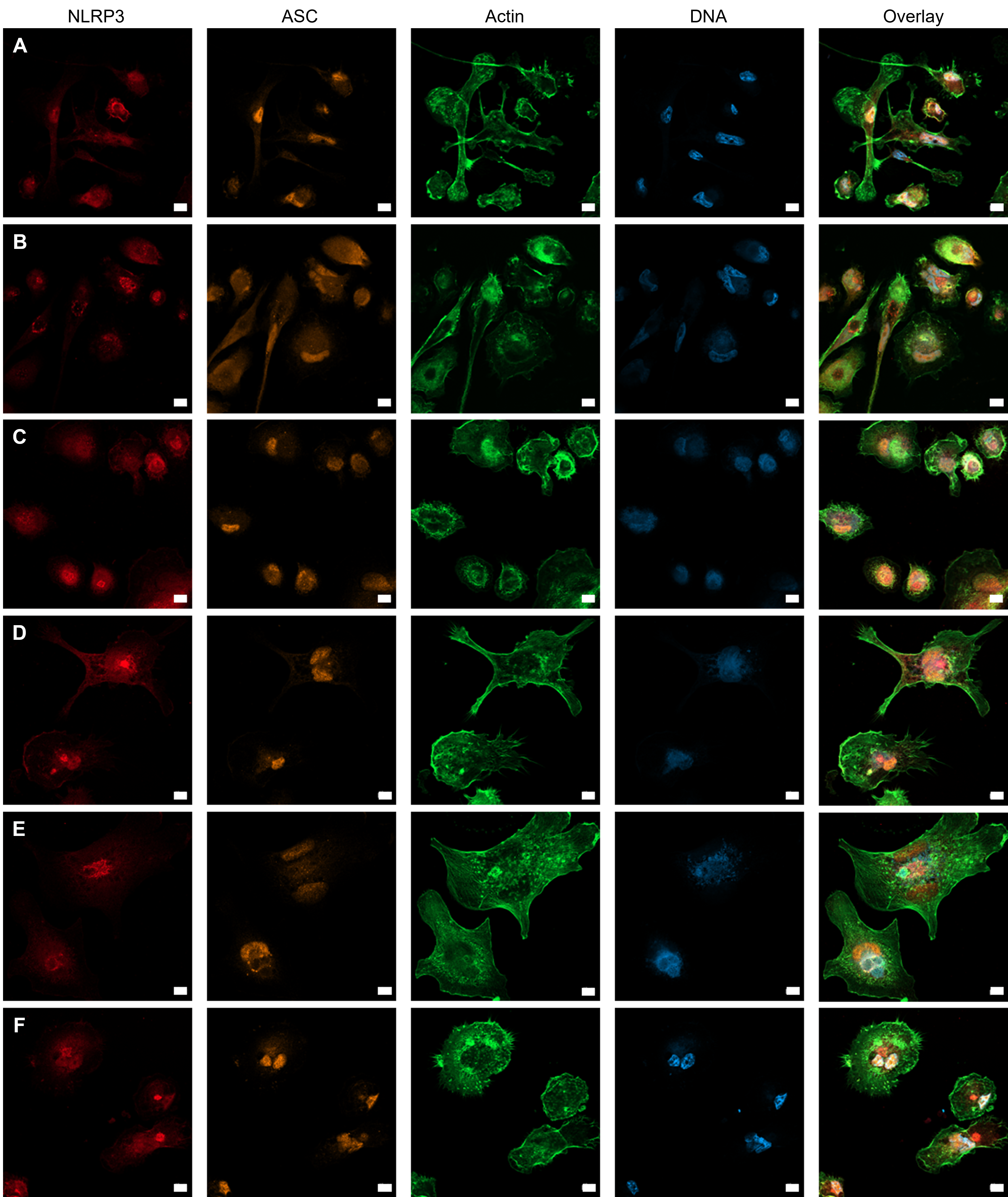

Supplement: Supplementary file 1 — Additional file 1: Supplementary Fig. 1: Confocal laser scanning micrographs of untreatedMΦ0,MΦ1 and MΦ1 cells incubated with 0.2 mg ml−1 ofnative β CD, or CD NPs prepared fromCDOC6,CDOC12, andCDSC6, visualized by staining of NLRP3, ASC, actin, and nuclei. Scale bars depict 20 µm. [file 11671_2024_4175_MOESM1_ESM.tif]

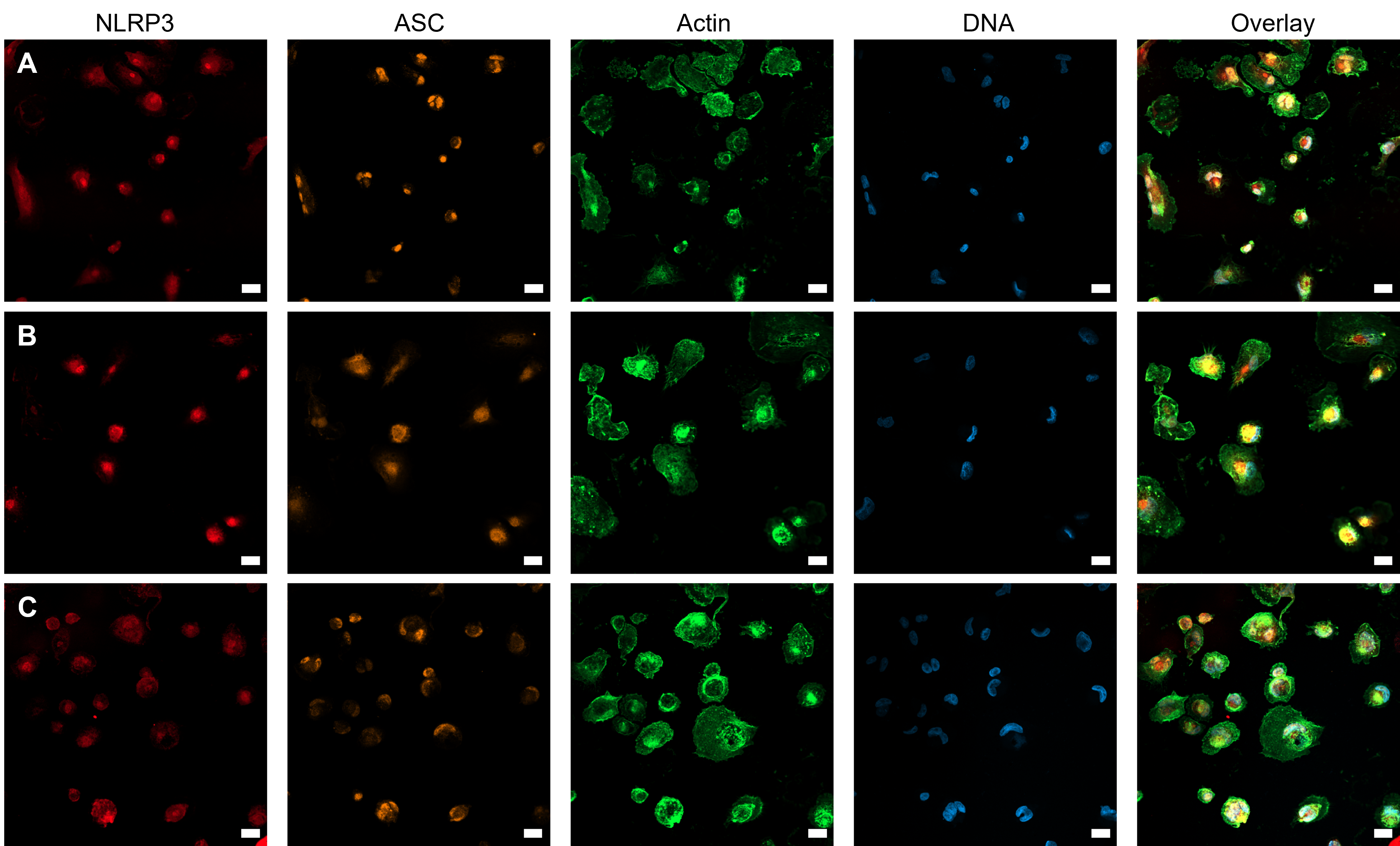

Supplement: Supplementary file 2 — Additional file 2: Supplementary Fig. 2: Confocal laser scanning micrographs of MΦ1 cells incubated with 0.1 mg ml−1 nanocarriers prepared fromCDOC6,CDOC12,CDSC6, visualized by NLRP3, ASC, actin, and nucleusstaining. Scale bars depict 20 µm. [file 11671_2024_4175_MOESM2_ESM.tif]
